# On Some Aspects of Memory

**Published:** 1901-01-19

**Authors:** John Aikman


### ON SOME ASPECTS OF MEMORY.

By JOHN AIKMAN, M.D.

### I.—PERSONAL AND RELATIVE—(*continued*).

There is no part of our bodies which does not receive impressions. We believe that it is chiefly our brain which records them. Men of science tell us that the grey coloured layer which forms the surface of our brain is the seat of the recording process. This grey layer is composed of cells, and the number of cells has been counted approximately; 600,000,000 is Sir S. Wilks' counting, and this estimate would allow of 50 cells per minute to be modified by impressions over a long life.

The most recent counting[1] is 920,000,000 grey or surface cells, and to the credit of the patience of her sex, be it recorded, this is the computation of a lady. No doubt the numbers vary in different brains, but there is one point of some interest with regard to the localisation of memory and thought in the surface cells of the brain which deserves notice.

You have all heard of water on the brain in children. You know that it is a very fatal disease. The water collects not only on the surface of the brain, but in its cavities. The stretching of the brain from water in its cavities is the most dangerous fact. But some children recover, and the brain which has been distended has a larger surface. The soft skull of the infant yields to the pressure of the fluid, and when it becomes bone in its entirety, it is larger than natural. What is the after history of these children? If the distension has been great, they approach or reach idiocy. But if the pressure has not been too great, the surface increases in extent. Mahomet, the prophet of Islam, has features which seem to indicate such a history, and he was an epileptic. Helmholtz had such a history, and the facts were verified after his death.[2] Is it to the increased surface of his brain that we owe the scientific suggestion which has drawn in his train the thought of Europe, and even now that he is dead, is so pregnant of further questions? Cuvier is a further example, Thackeray is another, and if I quoted more instances of children who had obviously survived infantile water on the brain of moderate amount, you would recognise that they claimed their place in the world's history as thinkers rathea than as physical actors.

[1] Brit. Med. Jour., Nov. 11, 1899, p. 1379. [2] Brit. Med. Jour., 1899, vol. i., p. 1180.

### II.—GEOGRAPHY AND TIDAL INFLUENCE.

BUT "he was only a poor philosopher who called the mind of children a blank page."[1] The scientist does not stop there. He tells us how the record is written, and something of the circumstances which make it deep or shallow.

Fifty brain cells, twenty-five brain cells, or seventy-five brain cells are ready to undergo modification during each minute of a long life. How? and why?

Let us ask the question of their history.

The grey brain cells of the infant are more or less egg-shaped. During growth large numbers of these cells become star-shaped, and many of the rays of these stars are found to have become continuous with the rays of other cells. In old age these star-like rays within become disconnected from the other cells, and acquire blunt club-like endings, not unlike the receivers in Marconi's system of wireless telegraphy. Such is the description of "no memory," "quick memory," and "blunted memory." The oval cell is the sensitised photographic plate; the stellate cell is the vivid image; the shrinking cell is the fading picture.

What dominates these changes?

There is a parallel in what is known as amœboid life. The word amœboid is derived from the Greek, and means to change. The class known as amœboid organisms change their shape to capture food. They extend their bodies towards nutritious particles, encircle them, and digest them.

The ovoid, or undeveloped, cells of the brain are believed to exist, and grow, under the conditions of amœboid life. The feelers which they put forth become contiguous to, or perhaps continuous with, the feelers of other cells. They establish at least such a contact as is made by the leaves of trees in a forest.[2] This contact creates a pathway. Along it a repetition of the force which produced it travels with least resistance. When the pathway is overcrowded, neighbouring cells feel the influence. The memory path is the easier one. It gains strength and permanence by repetition, and includes new cells when itself is too full. The picture is an ideal commonwealth. The first work falls upon the active members of the State. All surplus brings the previously unemployed to a position of usefulness.

Two conditions exist. The call for energy, and the food which supplies that energy. The infant's first thought is food. Before it has a thought the brain cells which govern the action of the heart are well developed. Next in order come those which recognise touch, then those which regulate breathing, and at the time of birth those which create appetite. That is the brain capital of a new-born infant. The amœboid cell represents the infancy of all living organisms. They search for their food, and the blood supplies it. The heart keeps the blood in movement. Strong elastic vessels which, we call arteries, carry the food-stored blood over the body. Fine hair-like vessels distribute it among the tissues. Large soft vessels, veins, drain the used-up blood, and return it for purification and reinforcement. It is from the fine hair-like vessels that the feeding is done. These vessels have a distinct wall when they spread themselves among the structures of the body. They have no recognisable wall in the brain substance. The wall of the hair-like vessels is needed to resist the pumping power of the heart, and the feeding is sufficiently rapid to renew the muscle which is used up by muscular movement. But thought is infinitely more rapid than muscular movement, and our brains have a special mechanism to supply the need.

If you place a glass tube in a tumbler of water, the water rises in the tube. If you lift the tube out, the water escapes. But if you place your finger on the top of the tube before lifting it out, the water does not escape. The same principle keeps the blood in the brain after the skull has become bone. The closed cavity of the skull resists the pumping power of the heart. It also hinders the escape of blood from the brain. The brain cells need themselves from a slowly-moving supply. They not only use food, they store it. A recent investigator, Nissl, has succeeded in staining the food substance stored in the brain cells. During health and life it is fluid, but it coagulates when disease is present, or life is absent. This stored food supplies all that is required for speedy action. How ready it is you will gather from a simple reflection. The blood is stored in the brain by the weight of the atmosphere upon the surface of the body. The surface of the body of an average-sized man measures about 17½ square feet.

[1] Tennyson's "Promise of May." [2] Mott, Croonian Lectures, 1900, Lancet, Vol. i. p. 1786.

(*To be continued.*)

